# Directed Evolution and Resolution Mechanism of 1, 3-Propanediol Oxidoreductase from *Klebsiella pneumoniae* toward Higher Activity by Error-Prone PCR and Bioinformatics

**DOI:** 10.1371/journal.pone.0141837

**Published:** 2015-11-03

**Authors:** Wei Jiang, Yuan Zhuang, Shizhen Wang, Baishan Fang

**Affiliations:** 1 Department of Chemical and Biochemical Engineering, College of Chemistry and Chemical Engineering, Xiamen University, Xiamen, Fujian, 361005, China; 2 The Key Lab for Synthetic Biotechnology of Xiamen City, Xiamen University, Xiamen, Fujian, 361005, China; 3 The Key Laboratory for Industrial Biotechnology of Fujian Higher Education, Hua Qiao University, Xiamen, Fujian, China; 4 The Key Laboratory for Chemical Biology of Fujian Province, Xiamen University, Xiamen, Fujian, 361005, China; The Scripps Research Institute, UNITED STATES

## Abstract

1, 3-propanediol oxidoreductase (PDOR) is a key enzyme in glycerol bioconversion to 1,3-propanediol (1, 3-PD) which is a valuable chemical and one of the six new petrochemical products. We used error-prone PCR and activity screening to identify mutants of *Klebsiella pneumoniae* (*K*. *pneumoniae*) PDOR with improved activity. The activity of one of the identified mutants, PDOR’-24, which includes a single mutation, A199S, was 48 U/mg, 4.9 times that of the wild-type enzyme. Molecular docking was performed to analyze the identified mutants; and amino acids S103, H271, N366, D106, N262 and D364 were predicted to bond with NADH. The origins of the improved activity of PDOR’-24, as well as three other mutants were analyzed by simulating the interaction mechanism of the mutants with the substrate and coenzyme, respectively. This research provides useful information about the use of safranine O plate screening for the directed evolution of oxidoreductases, identifies interesting sites for improving PDOR activity, and demonstrates the utility of using molecular docking to analyze the interaction mechanism of the mutants with the substrate and coenzyme, respectively.

## Introduction

1,3-Propanediol (1,3-PD) is a compound with two hydroxyl groups and can be used to synthesize polyester, polyether and polyurethane [1–3], etc. It is especially useful for the synthesis of polytrimethylene terephthalate (PTT), which is an excellent polyester compound with many outstanding properties. 1, 3-PD is produced by chemical methods and microbial conversion. Chemical synthesis is costly, energy consuming and produces pollution, which greatly limits the application of 1, 3-PD, while microbial conversion is a more attractive approach due to the mild reaction conditions needed, simple operation, high raw material to use ratio, and high product purity. The rate limiting step is the bioconversion of glycerol to 1, 3-PD catalyzed by the enzyme 1, 3-propanediol oxidoreductase (PDOR, EC 1.1.1.202), which directly hydrogenates 3-hydroxypropionaldehyde (3-HPA) to 1, 3-PD and is encoded by the *dha*T gene.

Enhancing the activity of PDOR is essential for the production of 1,3-PD. Although there has been much research about the PDORs from *Klebsiella pneumoniae* [4], *Lactobacillus reuteri* [5], *Lactobacillus buchneri*, *Lactobacillus brevis* [6], *Citrobacter freundii* [7], *Enterobacter agglomerans* [8], *Clostridium pasteurianum* [9] and *Clostridium butyricum* [10], little has been done concerning their structure-activity relationships and to our knowledge no effort to improve the activity of PDOR via directed evolution has been reported.

As a robust engineering technique, directed evolution can overcome the limitations of natural enzymes. This technique does not depend on a particular understanding of the relationship between the structure and function of enzyme; rather it relies on the straightforward powerful Darwinian principles of selection and mutation [11, 12]. Construction of a reasonable size library of mutated genes is the basis of protein directed evolution, while the establishment of efficient screening method with high sensitivity, low cost and less workload is the key to the success of directed evolution. Over the past few years, numerous screening methods such as the agar plate coating method, microporous plate suspension method, flow cytometry, and phage display, have been widely reported. Current error-prone PCR based on the inaccurate amplification of target genes is commonly used due to its efficiency in exploring sequence space [13–15]. By using directed evolution, it has been found that the catalytic efficiency of a mutant PC3 (D60N+Q103R+N218S) was 256 and 131 times higher than that of the wild type in 60% and 85% dimethylformamide (DMF), respectively [16]. After two rounds of directed evolution, the catalytic efficiency of the mutant PC3 was further improved to 471 times higher than that of the wild type in 60% DMF [17].

The PDOR from *K*. *pneumoniae* has high production capacity of 1, 3-PD and high expression levels in *E*. *coli* BL21 (DE3), making it an attractive candidate for random mutagenesis. In this work, we report improving the activity of the PDOR from *K*. *pneumoniae* using error-prone PCR and a safranine O plate screening method. After four rounds of error-prone PCR and one round of screening, we identified residue 199 as an interesting site of mutation and found mutants with a specific activity up to 4.9-fold higher than that of the wild-type. The potential mechanism for higher activity of PDOR’-24 and three other mutants was analyzed by simulating the interaction mechanism of the mutants with the coenzyme or substrate.

## Materials and Methods

### Reagents, strains and plasmids

All restriction enzymes, T4 DNA ligase, Ex *Taq* ^™^ DNA and Agarose Gel DNA Fragment Recovery Kit Ver.2.0 were purchased from TaKaRa Co. (Dalian, China) or Qiagen Co. (Germany). All the chemicals were of analytical grade and obtained from Sigma (United States). The strain *K*. *pneumoniae* DSM2026 was obtained from Dr. An-Ping Zeng (German Research Center for Biotechnology). *Escherichia coli* DH5α was used for general cloning and construction of mutagenesis library, while *E*. *coli* BL21 (DE3) was used as a host strain for protein expression. PlasmidpET-15b (+) was used for preparation of the mutant *dha*T library and expression of PDOR and its mutants. The components of filter medium include tryptone 6.0 g/L, yeast extract 3.0 g/L, NaCl 10.0 g/L, glycerine 18.4 g/L, safranine O 0.1 mmol/L, IPTG 1 mmol/L, agar powder 1.0% (W/V) pH was adjusted to 7.3, and 100μg/mL ampicillin was added before use.

### Cloning of *dha*T gene

The targeted *dha*T gene from the genomic DNA of *K*. *pneumoniae* was amplified by PCR using two primers (the forward primer K1: 5’-GGCCCATATGAGCTATCGTATGTTTGATTATC-3’, *Nde*I restriction site was underlined; and the reverse primer K2: 5’-ACTTGGATCCTCAGAATGCCTGGCGGAAAAT-3’, *Bam*HI restriction site is underlined) and Taq DNA polymerase using the following protocol: denaturation at 94°C for 5 min, 30 cycles of 30 s at 94°C, 1 min at 64°C, 2 min at 72°C, followed by a 10-min extension at 72°C. After PCR reaction, the amplified products were purified and digested with *Nde* I and *Bam*HI and then ligated using T4 DNA ligase into the *Nde* I-*Bam*HI restriction site of pET-15b to generate the plasmid pET-15b-*dha*T.

### Construction of error-prone library

The randomly mutated *dha*T library was constructed by error-prone PCR [18]. The error-prone PCR amplification system (20 μL), which was built after repeated comparison and experiment validation, was composed of Taq buffer containing 7 mM MgCl_2_, ~50 ng template plasmid pET-15b-*dha*T, 0.2 mM dATP and dGTP, 0.8 mM dTTP and dCTP, 0.5 mM Mn^2+^, 1.0 units of Taq DNA polymerase, and 37.5 pM primers (K1 and K2). The PCR reactions were carried out under the conditions described in the paragraph of the “Cloning of dhaT gene”, except the annealing temperature was 55°C. The strategy of continuous error-prone PCR was used, which utilized previously PCR amplification products were as the template for the next PCR amplification. Through continuous and repeatedly random mutations, single mutations accumulated to produce significant mutations. After four rounds of error-prone PCR, the amplified PCR products were digested by *Nde* I and *Bam*HI and ligated with plasmid pET-15b. Finally, the constructed plasmids were transformed into *E*. *coli* BL21 DE3 for expression.

### Library screening and expression of wild-type PDOR and mutant enzymes

**Fig 1:**
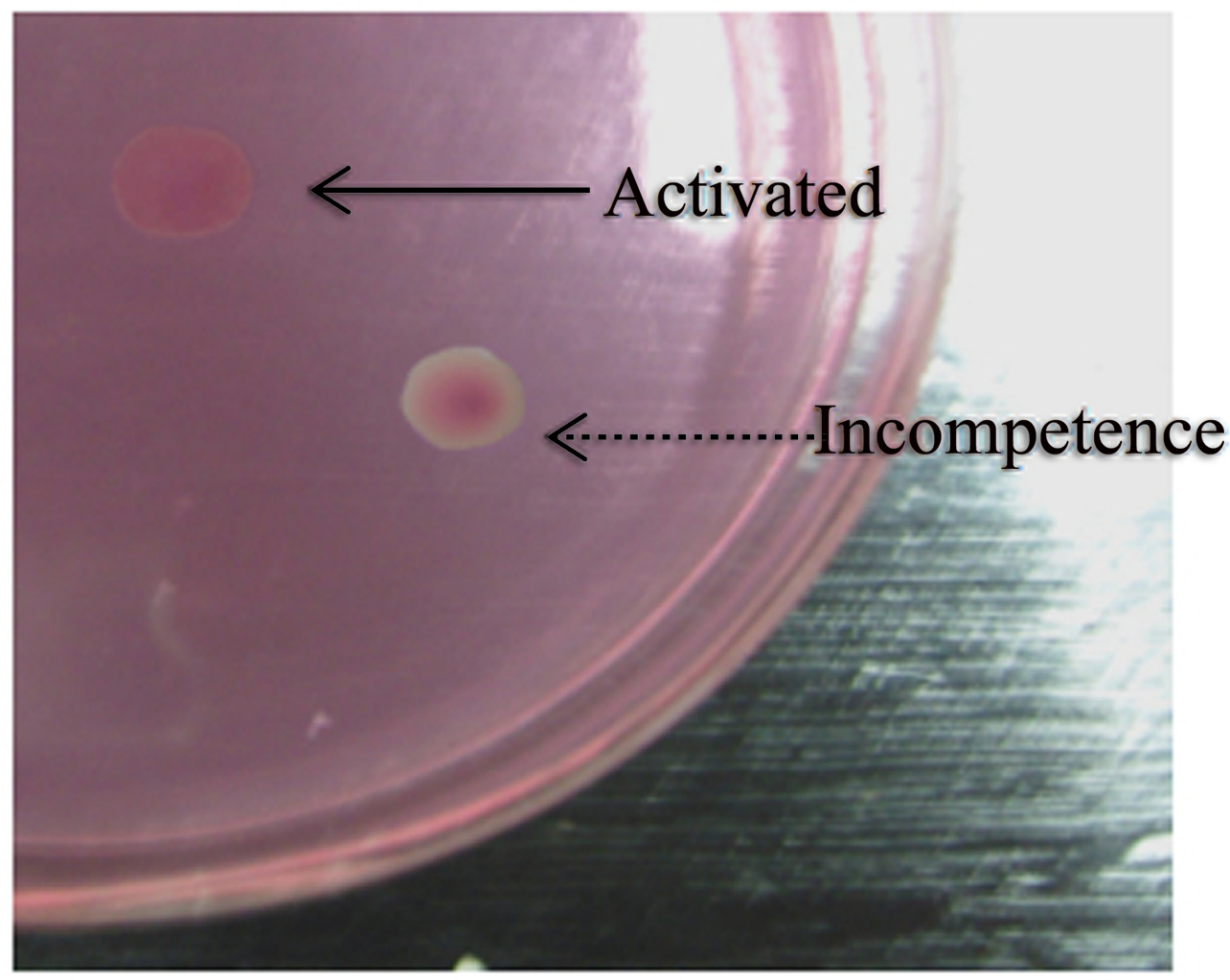
Identification of evolved mutants using the high throughput safranin O plates screening method. An example of an active and an inactive clone are shown.

The plasmids carrying the mutant genes of the *dha*T, were transformed into BL21 DE3 and the resulting strains were grown on the medium-ampicillin-safranine O screening plates for screening of mutants with increased activity. The plates were incubated under 37°C for about 12 h before further use. While PDOR oxidizes 1,3-PD to generate 3-HPA, NADH is produced which could increase the electric potential through the respiratory chain, produce strong proton flow, strengthen the *E*. *coli* cell’s intake of safranine O, and make the safranine O aggregate in *E*. *coli* cells [19]. Through the safranine O screening, the mutants with desired PDOR activity would show homogeneous peach-like pattern, while inactive cloning has a transparent ring around the colonies (Fig 1).

Single colonies from the mutant library were picked and grown in LB culture medium containing ampicillin (75μg mL^-1^) at 37°C. The overnight culture was then 1% v/v inoculated into fresh fermentation medium for cultivation at 30°C for 2 h. When the optical density (measured at 600 nm) of the culture reached 0.7–0.8, recombinant protein expression was induced by adding lactose to a final concentration of 2 g/L, followed by incubation at 25°C for 8h. Cells were then harvested for enzyme assay. The assay was conducted in 96-well plates using a microplate reader to yield mutant enzyme activity according to a previously reported protocol [19]. The enzyme was extracted by ultrasonic and purified by using Ni-IDA column (AKTA purifier, GE company).

### Enzymatic Activity Assay

Activity of PDOR was measured using 1,3-PD as the product following a method described by Ahrens et al. 1998 [20]. Activity of PDOR was determined spectrophotometrically at 340nm by measuring the initial rate of substrate-dependent NADH formation. The oxide type coenzyme NAD^+^ as the H receptors was reverted to the reduced coenzyme I NADH when PDOR oxidation of 1,3-PD to generate 3-HPA. NADH has the maximum absorption under 340 nm while NAD^+^ does not have its maximum absorption under 340 nm. As the absorbance is proportional to the concentration of NADH, the initial velocity method is adopted to determine the enzymatic activity of PDOR according to the increase of absorbance. The assay mixtures contained 30 mmol L^-1^ (NH_4_)_2_SO_4_, 1 μmol L^-1^ Fe(NH_4_)_2_(SO_4_)_2_, 0.1 mol L^-1^ 1,3-PD, 2 mmol L^-1^ NAD^+^, 0.1 mol L^-1^ potassium bicarbonate buffer (pH 9.5) and appropriate amount of enzyme in a final volume of 1.5mL. Cell-free extract was analyzed by dynamic absorbance measurement at 340nm in potassium carbonate buffer at pH 10.0 under 50°C. An enzyme activity unit (U) is defined as catalytic substrate conversion of 1 μmol 3-HPA by the required amount of the enzyme per minute.

### Molecular modeling and molecular docking

The position of the mutation site was indicated on the three-dimensional structure model of the PDOR, which was constructed from the known x-ray structure (PDB entry number 3BFJ) [21] using Swiss-Model (http://us.expasy.org/swissmod/SWISS-MODEL.htmL), a knowledge-based protein modeling tool [22]. Molecular docking method (MolSoft http://www.molsoft.com/) was implemented to simulate the position of the iron ion and the binding modes of PDOR with the substrate and coenzyme, respectively.

## Results

### Cloning of *dha*T gene

An 1173 bp DNA fragment, encoding PDOR, was obtained from *K*. *pneumoniae*. The predicted pI and molecular weight of the PDOR were 5.94 and 41.5 kD respectively. The isolated PDOR showed high similarity with PDOR from other sources at both nucleotide and amino acid levels. As PDOR has six consecutive β fold and Fe-NAD binding sites, it was considered as an ion activation dehydrogenase that belongs to NAD co-enzyme dependent glycerol dehydrogenase family type III. The isolated PDOR showed different degrees of identity with the PDOR from *K*. *pneumoniae* ATCC 25955 (98%, U30903.1), *C*. *freundii* (85%, U09771), *Chromobacterium violaceum* ATCC 12472 (86%, AE016919.1).

### Error-prone PCR mutant library

A PDOR mutant library was constructed using the error-prone PCR described in the materials and methods section. The screening of the random mutant library of *dha*T was performed using a high-throughput screening system that uses solid growth media containing safranine O. Through multiple rounds of screening, the PDOR’-24 mutant was isolated from over 7,000 clones in the error-prone library, and exhibited higher activity than the wild-type enzyme and other mutants. Sequencing analysis revealed that the PDOR’-24 mutant has a point mutation at the 199 site (A199S). For a better understanding of the relationship between the mutation site and the structure and function of PDOR, 3 largely negative mutants which have little activity, carried by *E*. *coli* BL21(DE3)/PET-15b-*dha*T’-39 (PDOR’-39, D94H), *E*. *coli* BL21(DE3)/PET-15b-*dha*T’-73(PDOR’-73, V155I) and *E*. *coli* BL21(DE3)/PET-15b-*dha*T’-85(PDOR’-85, D94H and also lacking the last three amino acids) were selected for further analysis and comparison.

### Enzyme assay of wild-type and mutant PDOR

**Fig 2:**
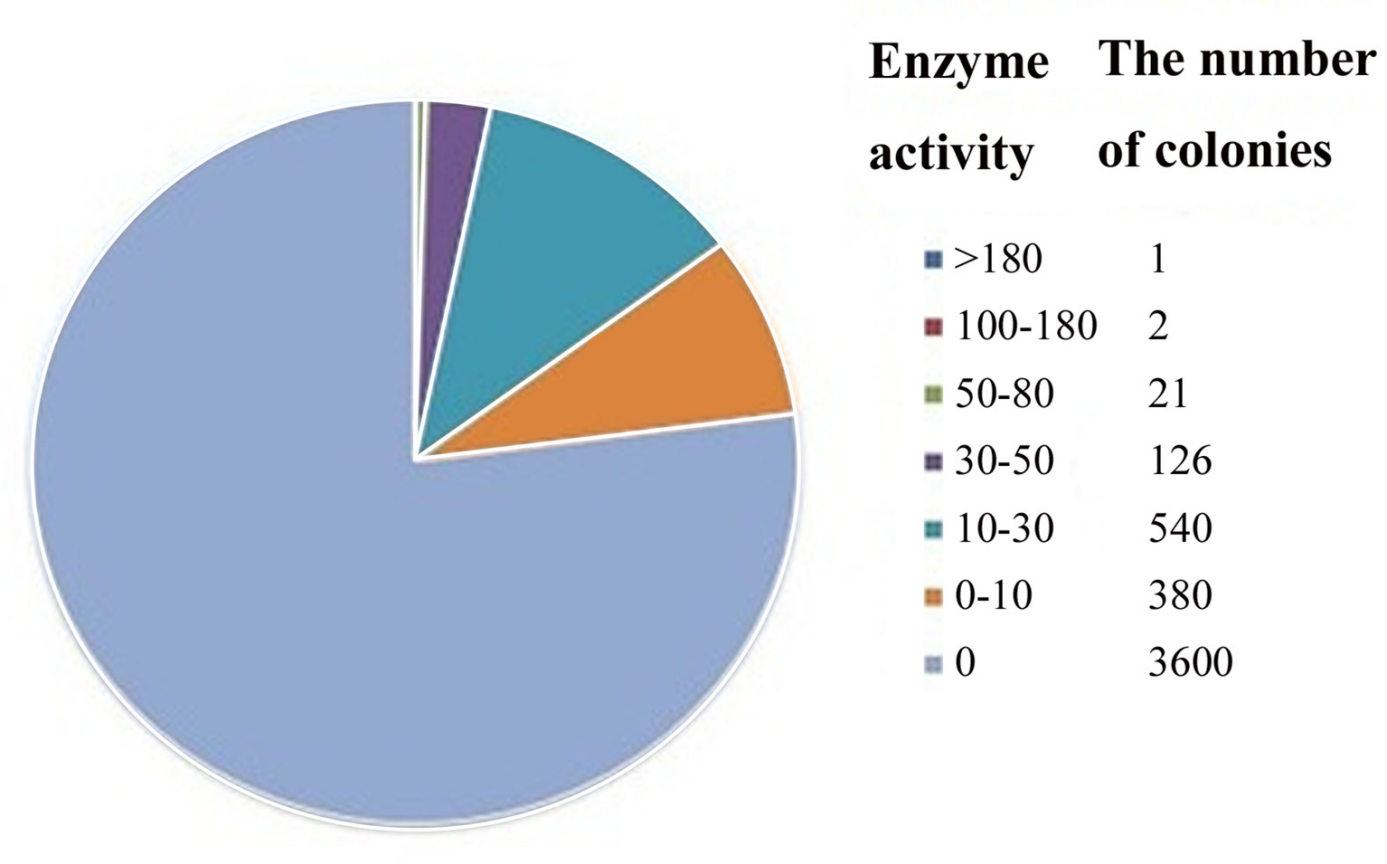
The pie chart of the distribution of PDOR’ activities and identified mutants.

After multiple rounds of screening, the mutants’ activities was increased from 2.9 U/mL to 182 U/mL. Distribution of mutants with varied enzyme activity are showed in Fig 2. Mutant PDOR’-24 showed 9.7 times higher activity than a mutant isolated from *K*. *pneumoniae* after optimization of medium and culture conditions [23], and 4.9 times higher activity than the wild-type enzyme. Specifically, enzyme activities of the PDOR’-24, PDOR’-39, PDOR’-73, PDOR’-85 and the wild-type were 48±0.5 U/mg, 2.3±0.1 U/mg, 8.1±0.6 U/mg, 0.81±0.1 U/mg and 9.9 ±0.3U/mg, respectively.

### The molecular docking of the mutants with the substrate and coenzyme

**Fig 3:**
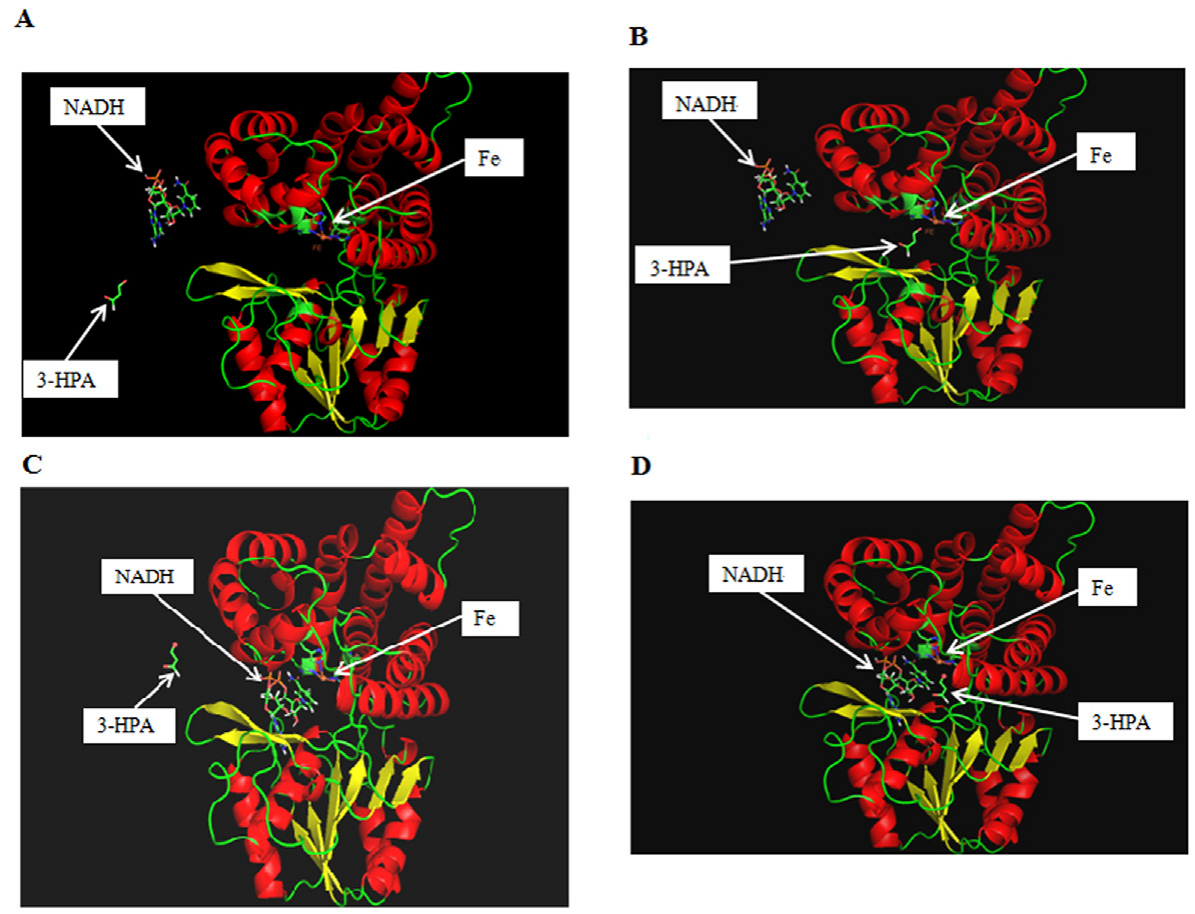
The bonding model of mutated PDOR’s with NADH or 3-HPA. A: before the NADH and 3-HPA bonding of mutated PDOR; B: bonding of mutated PDOR and 3-HPA; C: bonding of mutated PDOR and NADH; D: bonding of mutated PDOR, NADH and 3-HPA.

Five docking sites were obtained after analyzing the docking model of the PDOR and NADH by MolSoft software (Sections A and B in S2 File). Compared with the other four docking results, the best docking result had the lowest energy (-177.01 kJ/mol). Nine hydrogen bonds were formatted by the PDOR and NADH. Amino acids that docked with the NADH were S103, H271, N366, D106, N262 and D364. As the mutant proteins have similarity of between 99.0% and 99.7%, docking models of the NADH and PDOR were consistent. The binding sites and the relative position of the iron ion are presented in Fig 3.

**Fig 4:**
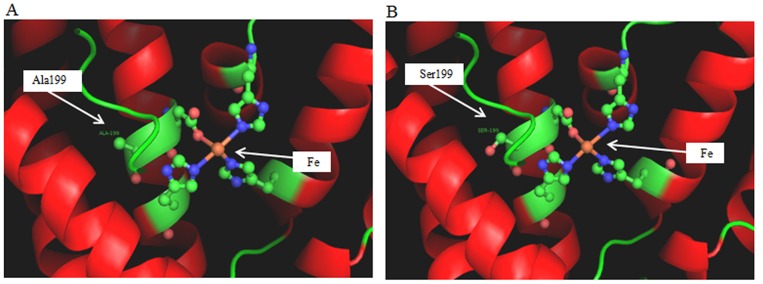
The three-dimensional structure of monomer of PDOR and PDOR’-24. A: Structure of monomer of PDOR without mutation; B: Structure of monomer of PDOR’-24 with mutation. These results were obtained by our own manipulations/docking.

After analysis of the docking model of the PDOR and 3-HPA in MolSoft software, five docking sites were obtained (Support information 2). Three of these docking sites with 3-HPA was at the 198^th^ aspartic acid whose od2 oxygen atoms were connected with the 3-HPA by a hydrogen bond. The binding sites and relative position of the iron ion is presented in Fig 3. A docking model of the A199S complex was constructed based on the homology model to identify the probable molecular basis for the enhancement of enzyme activity (Fig 4). The PDOR monomer contains 13 α helices and 8 β strands. The 199^th^ amino acid residue is located in the middle of the fifth α helix in the three-dimensional structure, while the iron binding site is at the 198^th^ amino acid, next to the aspartic acid residue. In order to understand the relationship between the mutation site and the structure and function of PDOR, the other 3 docking models of the PDOR’-39, D94H, PDOR’-73 and PDOR’-85 were also built and analyzed (Figures A-C in S1 File).

## Discussion

Currently, 1, 3-PD is mainly produced through enzymatic or cellular bioconversion from a renewable resource. It was demonstrated that the PDOR activity is the key factor for the product concentration of 1, 3-PD [24–28]. It is suggested that the research on PDOR for higher activity would be of great interest for improving the industrial production of 1, 3-PD and relevant future studies. As a well-established technique, directed evolution can overcome the limitations for improving the activity of natural enzymes which were used as industrial biocatalysts. In particular, this technique does not depend on a detailed understanding of the relationship between the structure and function of the enzymes; rather it utilizes the straightforward and powerful Darwinian principles of selection and mutation [29]. As far as we know, this is the first report to enhance the enzyme activity of PDOR by directed evolution while there are only few studies on the effect of amino acid residues on PDOR properties as well as the relationship between the structure and mutation sites. In this work, a safranine O plate screening methods was utilized to screen for PDOR with higher activity. Specifically, clones with PDOR activity formed uniformly pink colonies, while those without activity showed a transparent ring. The high-throughput screening system with high sensitivity, low cost of screening, and less workload could be of great research interest for directed evolution of other oxidoreductases such as glycerol dehydrogenase and formate dehydrogenase.

The 387-residue PDOR monomer, which is encoded by *dha*T from *K*. *pneumoniae*, can be divided into two functional structure domains: the N-terminal domain (residues 1 to 186) is responsible for the identification and combination of the substrates, and the C terminal domain (residues 189 to 387) is responsible for the combination of iron ions. All members of this family, the family III metal-dependent alcohol dehydrogenases, share a similar iron ion combination mode. This binding pattern involves three histidines (His267, His281 and His202) and one aspartic acid (Asp198) [21]. The possible binding site of PDOR and NADH or 3-HPA was predicted by MolSoft software. Nine hydrogen bonds were formed between the mutants and NADH, and the binding sites were S103, H271, N366, D106, N262 and D364, respectively. Using the od2 oxygen atoms, the 198^th^ aspartic acid was connected with the 3-HPA by a hydrogen bond.

After four-rounds of error-prone PCR, one round of screening and one additional round of re-screening, PDOR’-24 with the mutated site at the 199^th^ amino acid and higher activity was isolated. PDOR’-24 showed 9.7 times higher activity than that of a previously reported PDOR from *K*. *pneumoniae* after medium and culture conditions optimization [23]. Also, its activity was shown to be 4.9 times higher than wild-type enzyme. The analysis of the Ala-199-Ser mutation showed the impact on PDOR enzyme activity: by mutation of Ala 199 to Ser, there are some structural changes on gamma-C for more than one atom of oxygen which formed a hydroxyl with H. The distance of oxygen atoms of the hydroxyl and the active site of iron atoms is around 9Å, whereas the residues in 4Å space range of the oxygen atoms were Leu 200, Ile 227, Ala 250, Ser 251, Ala 254, and the residues in 5Å space range of the oxygen atoms were Ala 144, Gly 145, Gly 196, Asp 198, Leu 200, Ile 227, Ala 250, Ser 251 and Ala 254. As a result, the new oxygen on the Ala199-Ser199 lies in a hydrophobic environment. Without the mutation, the hydrophobic environment was relatively stable as it forms a rigid structure. However, the rigidity had a negative effect on stabilizing the active sites, as insufficient flexibility for the active sites clearly would reduce the binding with substrate and coenzyme NADH. For PDOR’-24, the additional hydroxyl group at the 199 site, likely somewhat increased the instability of hydrophobic microenvironment. The mutation adjacent to Asp-198 which was one of the four residues forming a covalent bond with Fe, generated an O atom only 9Å away from Fe atom. This mutation enhances the flexibility of a bipyramidal structure formed by Fe as a center and other 4 amino acids, which results in increase the activity space of the pyramidal structure, more space and probability to contact substrate, and more possibility to release from PDOR after product was formed. These effects may be the reasons of enhanced PDOR activities.

The decline of the activity of PDOR’-39, PDOR’-73 and PDOR’-85 could be attributed to the following reasons. First, PDOR’-39’s 94^th^ amino acid Asp, which is located between the third α helix and fourth β fold, was changed to His. As the size of histidine’s imidazole group is larger than that of Asp. This change could obstruct the binding of the benzene ring of the 98^th^ Tyr in the fourth β-pleated sheet, and thus reduce the stability of that β-pleated sheet. Second, PDOR’-73’s Ile puts one more methyl than Val at this position, which could extend toward opposite Fe from its own β-pleated sheet, and may also cause failure of combination of NADH and this enzyme, reducing the PDOR activity. PDOR’-85 has both the mutations belonging to PDOR’-39 and its own- nonsense mutation at C terminal which causes termination of 3 amino acids. As a result, PDOR activities of this mutant were also the lowest among all the mutant strains tested in this whole experiment. Further exploration of the structure-activity relationship of PDOR is needed due to our relatively poor understanding of this; and the results of this study could pave the way for future studies in this direction.

In this work, we demonstrated that the combination of directed evolution and a safranine O plate screening method could be used to improve PDOR enzyme activity. The activity of the PDOR’-24 mutant was 4.9-times higher than that of wild type. The interactions between or H-bonding interactions between amino acid residues, S103, H271, N366, D106, N262 and D364, and NADH were predicted to be involved in the enzyme activity increase. The reasons for improvement of activity with PDOR’-24 and three other mutants were investigated by simulating the binding mechanism of the mutants with the substrate and coenzyme, respectively, as well as the structural changes of the mutant site. This research reports the method of using the safranine O plate screening method for directed evolution of an oxidoreductase, and investigated the potential protein sites for improving the enzyme activity, using the molecular docking analysis.

## Supporting Information

S1 File Three Supporting Figures. Figure A. The three-dimensional structure of monomer of PDOR and PDOR’-39. A: Structure of monomer of PDOR without mutation; B: Structure of monomer of PDOR’-39 with mutation. Figure B. The three-dimensional structure of monomer of PDOR and PDOR’-73. A: Structure of monomer of PDOR without mutation; B: Structure of monomer of PDOR’-73 with mutation. Figure C. The three-dimensional structure of monomer of PDOR and PDOR’-85. A: Structure of monomer of PDOR without mutation; B: Structure of monomer of PDOR’-85 with mutation.
(DOC)

S2 File Results of the docking. Section A. Five results of the docking site of PDOR and NADH. Section B. Five results of the docking site of PDOR and 3-HPA.
(DOC)

## References

[1] Biebl H, Menzel K, Zeng A-P, Deckwer W-D. Microbial production of 1, 3-propanediol. Appl Microbiol Biotech. 1999;52(3):289–97. 10.1007/s002530051523 10531640

[2] Witt U, Müller RJ, Augusta J, Widdecke H, Deckwer WD. Synthesis, properties and biodegradability of polyesters based on 1, 3-propanediol. Macromol Chem Phys. 1994;195(2):793–802.

[3] KD A. Preparation of 1,3-propanediol used in polyester production [P]. US Pat. 1998; US 5 777 182.

[4] Johnson E, Lin E. *Klebsiella pneumoniae* 1, 3-propanediol: NAD^+^ oxidoreductase. J Bacteriol 1987;169(5):2050–4. 3553154 10.1128/jb.169.5.2050-2054.1987 PMC212087

[5] Talarico TL, Dobrogosz WJ. Purification and characterization of glycerol dehydratase from *Lactobacillus reuteri* . Appl Environ Microbiol. 1990;56(4):1195–7. 16348166 10.1128/aem.56.4.1195-1197.1990 PMC184372

[6] Veiga-Da-Cunha M, Foster MA. 1, 3-Propanediol: NAD^+^ oxidoreductases of *Lactobacillus brevis* and *Lactobacillus buchneri* . Appl Env Microbiol 1992;58(6):2005–10. 1622279 10.1128/aem.58.6.2005-2010.1992 PMC195718

[7] Daniel R, Boenigk R, Gottschalk G. Purification of 1, 3-propanediol dehydrogenase from *Citrobacter freundii* and cloning, sequencing, and overexpression of the corresponding gene in *Escherichia coli* . J Bacteriol. 1995;177(8):2151–6. 7721705 10.1128/jb.177.8.2151-2156.1995 PMC176860

[8] Barbirato F, Larguier A, Conte T, Astruc S, Bories A. Sensitivity to pH, product inhibition, and inhibition by NAD^+^ of 1, 3-propanediol dehydrogenase purified from Enterobacter agglomerans CNCM 1210. Arch Microbiol. 1997;168(2):160–3. 9238108 10.1007/s002030050482

[9] Luers F, Seyfried M, Daniel R, Gottschalk G. Glycerol conversion to 1, 3-propanediol by *Clostridium pasteurianum*: cloning and expression of the gene encoding 1, 3-propanediol dehydrogenase. FEMS Microbiol Lett. 1997;154(2):337–45. 9311132 10.1111/j.1574-6968.1997.tb12665.x

[10] Malaoui H, Marczak R. Purification and characterization of the 1-3-propanediol dehydrogenase of *Clostridium butyricum* E5. Enzyme Microb Technol. 2000;27(6):399–405. 10938419 10.1016/s0141-0229(00)00219-2

[11] Stemmer WP. DNA shuffling by random fragmentation and reassembly: in vitro recombination for molecular evolution. Proc Natl Acad Sci U S A. 1994;91(22):10747–51. 7938023 10.1073/pnas.91.22.10747 PMC45099

[12] Leisola M, Turunen O. Protein engineering: opportunities and challenges. Appl Microbiol Biotechnol. 2007;75(6):1225–32. 17404726 10.1007/s00253-007-0964-2

[13] Wong TS, Tee KL, Hauer B, Schwaneberg U. Sequence saturation mutagenesis (SeSaM): a novel method for directed evolution. Nucleic Acids Res. 2004;32(3):e26–e. 14872057 10.1093/nar/gnh028 PMC373423

[14] Lin L, Meng X, Liu P, Hong Y, Wu G, Huang X, et al Improved catalytic efficiency of Endo-β-1, 4-glucanase from *Bacillus subtilis* BME-15 by directed evolution. Appl Microbiol Biotechnol. 2009;82(4):671–9. 10.1007/s00253-008-1789-3 19050861

[15] Stephens DE, Singh S, Permaul K. Error-prone PCR of a fungal xylanase for improvement of its alkaline and thermal stability. FEMS Microbiol Lett. 2009;293(1):42–7. 10.1111/j.1574-6968.2009.01519.x 19220468

[16] Chen K, Arnold FH. Enzyme engineering for nonaqueous solvents: random mutagenesis to enhance activity of subtilisin E in polar organic media. Nat Biotechnol. 1991;9(11):1073–7. 10.1038/nbt1191-1073 1367624

[17] Chen K, Arnold FH. Tuning the activity of an enzyme for unusual environments: sequential random mutagenesis of subtilisin E for catalysis in dimethylformamide. Proc Natl Acad Sci U S A. 1993;90(12):5618–22. 8516309 10.1073/pnas.90.12.5618 PMC46772

[18] Johannes TW, Zhao H. Directed evolution of enzymes and biosynthetic pathways. Curr Opin Microbiol. 2006;9(3):261–7. 16621678 10.1016/j.mib.2006.03.003

[19] van Loo B, Spelberg JHL, Kingma J, Sonke T, Wubbolts MG, Janssen DB. Directed Evolution of Epoxide Hydrolase from A. radiobacter toward Higher Enantioselectivity by Error-Prone PCR and DNA Shuffling. Chem Biol. 2004;11(7):981–90. 15271356 10.1016/j.chembiol.2004.04.019

[20] Ahrens K, Menzel K, Zeng AP, Deckwer WD. Kinetic, dynamic, and pathway studies of glycerol metabolism by *Klebsiella pneumoniae* in anaerobic continuous culture: III. Enzymes and fluxes of glycerol dissimilation and 1, 3-propanediol formation. Biotechnol Bioeng 1998;59(5):544–52. 10099370 10.1002/(sici)1097-0290(19980905)59:5<544::aid-bit3>3.0.co;2-a

[21] Marçal D, Rêgo AT, Carrondo MA, Enguita FJ. 1, 3-Propanediol dehydrogenase from *Klebsiella pneumoniae*: decameric quaternary structure and possible subunit cooperativity. J Bacteriol. 2009;191(4):1143–51. 10.1128/JB.01077-08 19011020 PMC2631990

[22] Peitsch M. Protein modeling by E-mail. Bio/technology. 1995;13:658–60.

[23] Hongwen C, Baishan F, Zongding H. Optimization of process parameters for key enzymes accumulation of 1, 3-propanediol production from *Klebsiella pneumoniae* . Biochemical Engineering Journal. 2005;25(1):47–53.

[24] Nakamura CE, Whited GM. Metabolic engineering for the microbial production of 1, 3-propanediol. Curr Opin Biotechnol. 2003;14(5):454–9. 14580573 10.1016/j.copbio.2003.08.005

[25] Cao Y, Xia Q, Fang B. Optimization of expression of dhaT gene encoding 1, 3-propanediol oxidoreductase from Klebsiella pneumoniae in *Escherichia coli* using the methods of uniform design and regression analysis. J Chem Technol Biotechnol. 2006;81(1):109–12.

[26] Zhao L, Ma X, Zheng Y, Zhang J, Wei G, Wei D. Over-expression of glycerol dehydrogenase and 1, 3-propanediol oxidoreductase in *Klebsiella pneumoniae* and their effects on conversion of glycerol into 1, 3-propanediol in resting cell system. J Chem Technol Biotechnol. 2009;84(4):626–32.

[27] Zhuge B, Zhang C, Fang H, Zhuge J, Permaul K. Expression of 1, 3-propanediol oxidoreductase and its isoenzyme in *Klebsiella pneumoniae* for bioconversion of glycerol into 1, 3-propanediol. Appl Microbiol Biotechnol. 2010;87(6):2177–84. 10.1007/s00253-010-2678-0 20499228

[28] Li W, Ng I-S, Fang B, Yu J, Zhang G. Codon optimization of 1, 3-propanediol oxidoreductase expression in *Escherichia coli* and enzymatic properties. Electronic Journal of Biotechnology. 2011;14(4):7-.

[29] Zhang Z-G, Yi Z-L, Pei X-Q, Wu Z-L. Improving the thermostability of *Geobacillus stearothermophilus* xylanase XT6 by directed evolution and site-directed mutagenesis. Bioresour Technol. 2010;101(23):9272–8. 10.1016/j.biortech.2010.07.060 20691586

