# Supplementary material for: Directed Evolution and Resolution Mechanism of 1, 3-Propanediol Oxidoreductase from Klebsiella pneumoniae toward Higher Activity by Error-Prone PCR and Bioinformatics
Source: PLoS One. 2015 Nov 3;10(11):e0141837. doi: 10.1371/journal.pone.0141837 (PMC4631369; doi:10.1371/journal.pone.0141837)
Supplement: S1 File — Figure A. The three-dimensional structure of monomer of PDOR and PDOR’-39. A: Structure of monomer of PDOR without mutation; B: Structure of monomer of PDOR’-39 with mutation. Figure B. The three-dimensional structure of monomer of PDOR and PDOR’-73. A: Structure of monomer of PDOR without mutation; B: Structure of monomer of PDOR’-73 with mutation. Figure C. The three-dimensional structure of monomer of PDOR and PDOR’-85. A: Structure of monomer of PDOR without mutation; B: Structure of monomer of PDOR’-85 with mutation. (DOC) [file pone.0141837.s001.doc]

**S1 File. Three Supporting Figures.**

Figure A. The three-dimensional structure of monomer of PDOR and PDOR’-39. A: Structure of monomer of PDOR without mutation; B: Structure of monomer of PDOR’-39 with mutation.

Figure B. The three-dimensional structure of monomer of PDOR and PDOR’-73. A: Structure of monomer of PDOR without mutation; B: Structure of monomer of PDOR’-73 with mutation.

Figure C. The three-dimensional structure of monomer of PDOR and PDOR’-85. A: Structure of monomer of PDOR without mutation; B: Structure of monomer of PDOR’-85 with mutation.

**
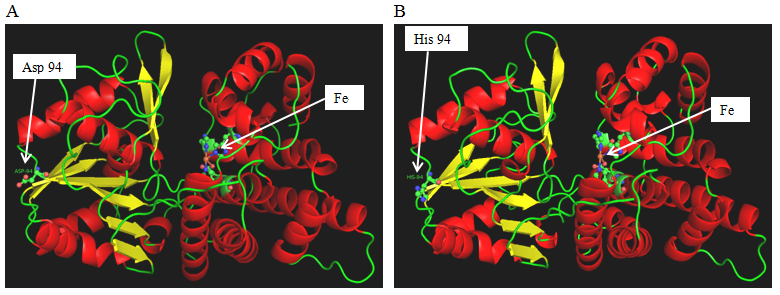
**

**Figure A The three-dimensional structure of monomer of the PDOR and PDOR’-39**

**
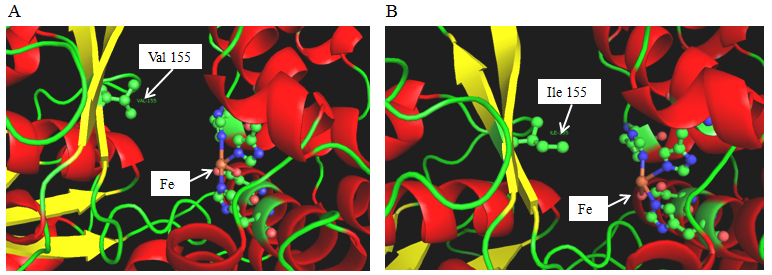
**

**Figure B The three-dimensional structure of monomer of the PDOR and PDOR’-73**

**
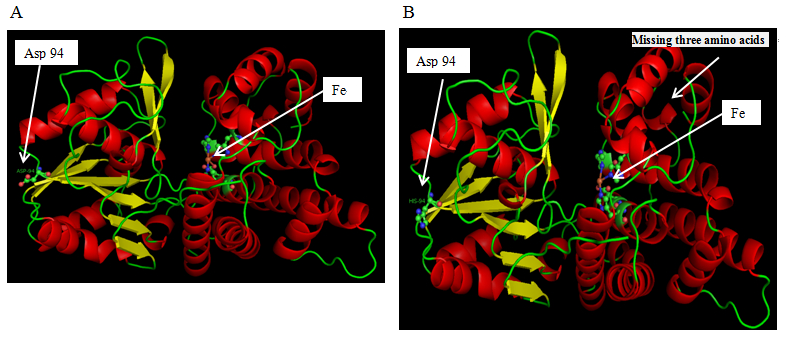
**

**Figure C Figure The three-dimensional structure of monomer of the PDOR and PDOR’-85**
