# Supplementary material for: Directed Evolution and Resolution Mechanism of 1, 3-Propanediol Oxidoreductase from Klebsiella pneumoniae toward Higher Activity by Error-Prone PCR and Bioinformatics
Source: PLoS One. 2015 Nov 3;10(11):e0141837. doi: 10.1371/journal.pone.0141837 (PMC4631369; doi:10.1371/journal.pone.0141837)
Supplement: S2 File — Section A. Five results of the docking site of PDOR and NADH. Section B. Five results of the docking site of PDOR and 3-HPA. (DOC) [file pone.0141837.s002.doc]

**S2 File. Results of the docking.**

Section A. Five results of the docking site of PDOR and NADH

Section B. Five results of the docking site of PDOR and 3-HPA

**Section A Five results of the docking site of PDOR and NADH**

Conf 1 of 95 energy=-177.01:

HBOND m S103 hn -- m M n2 : 2.23 A

HBOND m S103 hg -- m M n2 : 1.69 A

HBOND m H271 hd1 -- m Mo12 : 2.72 A

HBOND m N366 hd22 --m Mo8 : 2.55 A

HBOND m D106 od2 -- m M h03 : 2.17 A

HBOND m D106 od2 -- m M h01 : 2.51 A

HBOND m N262 od1 -- m M h72 : 2.48 A

HBOND m D364 od1 -- m M h04 : 2.36 A

HBOND m D364 od2 -- m M h04 : 2.35 A

total number of receptor-ligand H-bonds: 9

next/ back/ jump/ stop/ keep & stop (n/b/j/s/k) ? : n

Conf 2 of 95 energy=-175.94:

HBOND m N75 hn -- m M o1 : 2.11 A

HBOND m S103 hg -- m M o2 : 2.03 A

HBOND m K164 hz1 -- m Mo7 : 2.55 A

HBOND m K164 hz2 -- m M o7 : 2.16 A

HBOND m N366 hd22 -- m M o11 : 2.66 A

HBOND m N75 od1 -- m M h31 : 2.29 A

HBOND m H153 nd1 -- m M h05 : 2.43 A

HBOND m D278 o -- m M h02 : 2.56 A

HBOND m D278 o -- m M h52 : 2.65 A

HBOND m M279 o -- m M h02 : 2.74 A

total number of receptor-ligand H-bonds: 10

next/ back/ jump/ stop/ keep & stop (n/b/j/s/k) ? : n

Conf 3 of 95 energy=-174.68:

HBOND m N75 hn -- m M o3 : 2.74 A

HBOND m S103 hg -- m M o3 : 2.32 A

HBOND m S103 hg -- m M o2 : 2.15 A

HBOND m K164 hz2 -- m M o7 : 2.53 A

HBOND m G282 hn -- m M n4 : 2.39 A

HBOND m N366 hd22 -- m M o11 : 2.38 A

HBOND m H153 nd1 -- m M h05 : 2.23 A

HBOND m A194 o -- m M h52 : 2.69 A

HBOND m D198 od2 -- m M h02 : 2.23 A

total number of receptor-ligand H-bonds: 9

next/ back/ jump/ stop/ keep & stop (n/b/j/s/k) ? : n

Conf 4 of 95 energy=-174.46:

HBOND m S103 hn -- m M o3 : 2.71 A

HBOND m S103 hn -- m M o2 : 2.06 A

HBOND m S103 hg -- m M o2 : 1.64 A

HBOND m K164 hz2 -- m M o6 : 2.58 A

HBOND m K164 hz3 -- m M o6 : 2.45 A

HBOND m H281 hn -- m M n3 : 2.15 A

HBOND m H281 hd1 -- m M o7 : 2.74 A

HBOND m H153 nd1 -- m M h05 : 2.71 A

HBOND m D364 od2 -- m M h06 : 2.60 A

total number of receptor-ligand H-bonds: 9

next/ back/ jump/ stop/ keep & stop (n/b/j/s/k) ? : n

Conf 5 of 95 energy=-174.35:

HBOND m N75 hn -- m M o2 : 2.46 A

HBOND m S103 hg -- m M o3 : 2.64 A

HBOND m S103 hg -- m M o2 : 2.29 A

HBOND m G282 hn -- m M n4 : 2.67 A

HBOND m N366 hd22 -- m M o11 : 1.60 A

HBOND m S103 og -- m M h03 : 2.14 A

HBOND m H153 nd1 -- m M h05 : 2.35 A

HBOND m D198 od2 -- m M h02 : 2.58 A

total number of receptor-ligand H-bonds: 8

next/ back/ jump/ stop/ keep & stop (n/b/j/s/k) ? : n

**Section B Five results of the docking site of PDOR and 3-HPA**

Conf 1 of 24 energy=-30.45:

HBOND m D198 od2 -- m M h01 : 2.06 A

total number of receptor-ligand H-bonds: 1

next/ back/ jump/ stop/ keep & stop (n/b/j/s/k) ? : n

Conf 2 of 24 energy=-29.11:

HBOND m D198 od2 -- m M h01 : 2.24 A

total number of receptor-ligand H-bonds: 1

next/ back/ jump/ stop/ keep & stop (n/b/j/s/k) ? : n

Conf 3 of 24 energy=-28.95:

HBOND m H153 he2 -- m M o1 : 2.69 A

HBOND m N366 hd22 -- m M o2 : 1.84 A

HBOND m N262 od1 -- m M h01 : 2.32 A

total number of receptor-ligand H-bonds: 3

next/ back/ jump/ stop/ keep & stop (n/b/j/s/k) ? : n

Conf 4 of 24 energy=-28.85:

HBOND m D198 od2 -- m M h01 : 2.28 A

total number of receptor-ligand H-bonds: 1

next/ back/ jump/ stop/ keep & stop (n/b/j/s/k) ? : n

Conf 5 of 24 energy=-28.50:

HBOND m N366 hd22 -- m M o1 : 1.80 A

HBOND m N366 od1 -- m M h01 : 2.71 A

total number of receptor-ligand H-bonds: 2

next/ back/ jump/ stop/ keep & stop (n/b/j/s/k) ? : n

Conf 6 of 24 energy=-28.44:

HBOND m H271 ne2 -- m M h01 : 2.41 A

total number of receptor-ligand H-bonds: 1

next/ back/ jump/ stop/ keep & stop (n/b/j/s/k) ? : n

Conf 7 of 24 energy=-27.58:

HBOND m N366 hd22 -- m M o2 : 1.90 A

HBOND m H202 ne2 -- m M h01 : 2.57 A

total number of receptor-ligand H-bonds: 2

next/ back/ jump/ stop/ keep & stop (n/b/j/s/k) ? : n

Conf 8 of 24 energy=-27.43:

HBOND m D198 od2 -- m M h01 : 2.28 A

total number of receptor-ligand H-bonds: 1

next/ back/ jump/ stop/ keep & stop (n/b/j/s/k) ? : n
